# Environmental and economic data on energy efficiency measures for residential buildings

**DOI:** 10.1016/j.dib.2019.104905

**Published:** 2019-11-28

**Authors:** Delia D'Agostino, Danny Parker, Paco Melià

**Affiliations:** a European Commission, Joint Research Centre (JRC), Ispra, Italy; b Florida Solar Energy Center, University of Central Florida, USA; c Dipartimento di Elettronica, Informazione e Bioingegneria, Politecnico di Milano, Milano, Italy

**Keywords:** Energy efficiency, Technology measures, Building simulation, Embodied energy

## Abstract

This data article refers to the paper “Environmental and economic implications of energy efficiency in new residential buildings: a multi-criteria selection approach” [1]. The reported data deal with energy efficiency measures for residential buildings. This paper provides environmental and economic data related to envelope, appliances, and system measures. The calculations of the embodied energy associated with different building parts are included in the provided data. Available data relate to investment costs, lifetime, payback, net present value, embodied and operational energy, CO_2_ emissions, electricity and gas savings derived for each different energy efficiency measure. These data can be used to select the most suitable measures for residential buildings.

**Table:** Specification Table

Subject	*Energy*
Specific subject area	*Energy efficiency measures for residential buildings*
Type of data	*Datasheets*
How data were acquired	*Data were processed in BeOpt and Excel*
Data format	*Raw*
Parameters for data collection	*Data on costs, lifetime, payback, embodied energy, operational energy, electricity and gas savings for different energy efficiency measures*
Description of the data collection	*Data were collected from different sources* [[2], [3], [4], [5], [6], [7], [8], [9], [10]]
Data source location	*Milan (Italy)*
Data accessibility	*Data are provided in supplementary materials directly with this article*
Related research article	*Delia D'Agostino, Danny Parker, Paco Melià, Environmental and economic implications of energy efficiency in new residential buildings: a multi-criteria selection approach, Energy Strategy Reviews 26 (2019) 100412,*https://doi.org/10.1016/j.esr.2019.10041

**Table:** 

**Value of the Data** • The data support the selection of energy efficiency measures in residential buildings; • Environmental and economic data related to energy efficiency measures can be used to guide the design a new building; • The data show the energy savings and CO_2_ emissions associated to different envelope, appliances, and system measures; • The data derive costs, lifetime, payback, embodied and operational energy for several energy efficiency measures; • The data can be useful for the development of future energy policies, comparison with other building types and selection approach, or further analysis.

## 1 Data description

**Fig. 1:**
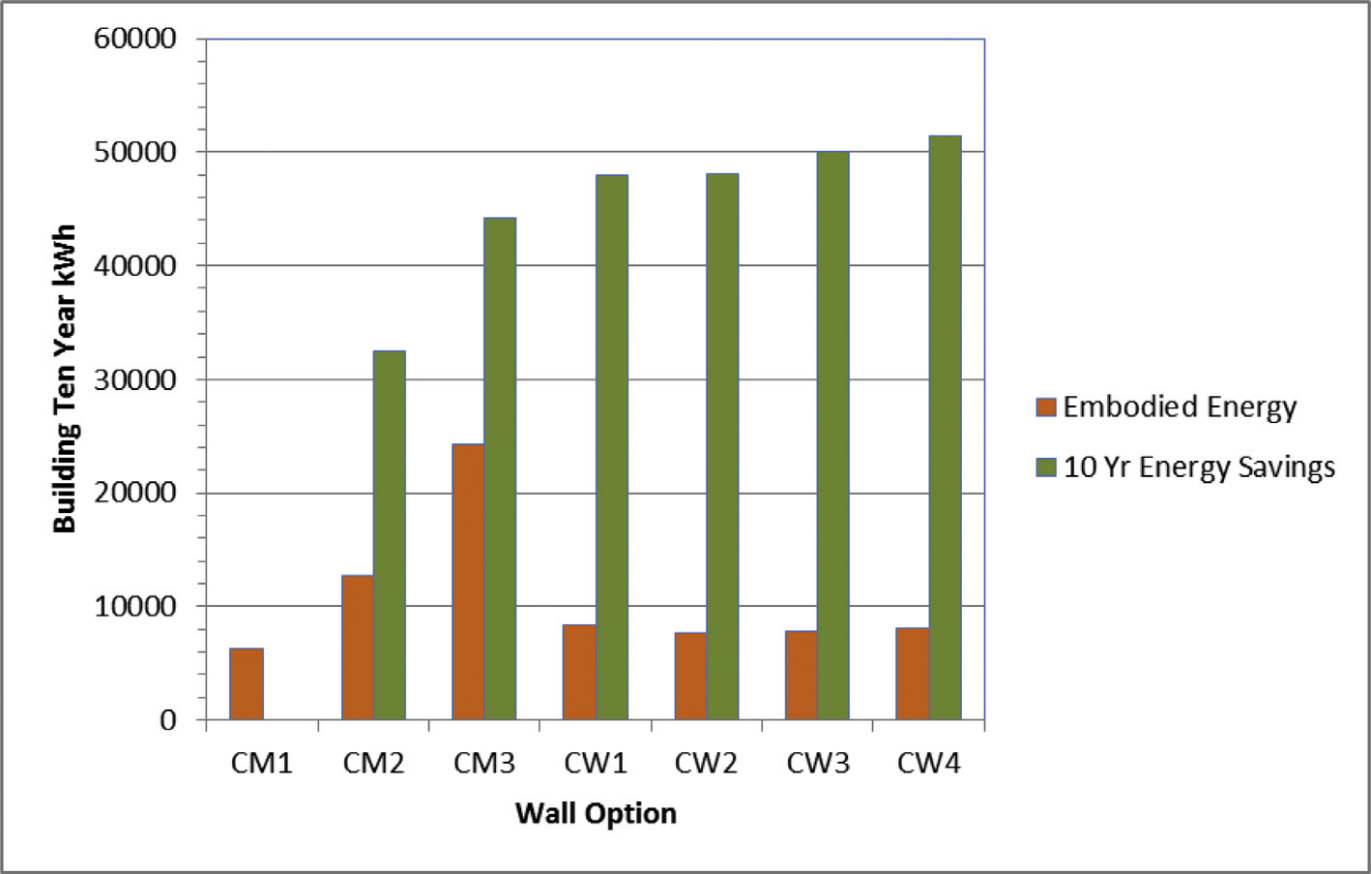
Comparative 10 year incremental embodied energy and energy savings for key wall options. CM1-CM3 are masonry wall systems (CM1 is the base case). CW1-4 are wood-framed walls with increasing cellulose cavity insulation showing lower embodied energy along with good long-term site energy savings.

The specific data are rendered as a detailed spreadsheet documenting the relevant calculation processes. This includes information on each evaluated measure or option, such as insulation, appliances and equipment. For each option, the estimated overall and incremental costs are provided, along with measure lifetime and physical characteristics. The embodied energy for complex specific wall constructions are also provided (see Fig. 1). This documents the detailed estimation of embodied energy, considering material density, volume, weight and type. The simulated measure savings in electricity and natural gas for each option are given along with associated CO_2_ emission reductions as well as the lifetime monetary energy savings and simple payback. Other columns in the data report on the economic performance of each evaluated option as well as the raw simulation results from evaluating each competing option in the assessment.

The choice of the technologies to be implemented in buildings is not an easy task at the design stage [11]. Many efficiency measures can reduce the operational energy required to heat, cool, heat water and run appliances in a building, such as foam insulations or advanced windows using many plastics. However, these measures can substantially increase the embodied energy required to manufacture the materials and equipment necessary to assemble the building [12]. Compared with operational energy, a higher uncertainty involves the evaluation of embodied energy, which is usually not considered [13]. This paper takes into account the data of the energy needed to locate, refine, manufacture and install the different efficiency measures in addition to the energy saved through those measures (both electricity and gas). Details on the calculations are available in Section 2.3 of [1]. Additionally, data on investments costs, net present value, CO_2_ emissions are also derived.

A new residential building located in Milan (Italy) is chosen in a case study that aims at the minimization of embodied energy and investment costs, and maximization of electricity and gas savings associated with each efficiency measure. The approach allows a comparison of alternative technologies to be potentially implemented using environmental and economic criteria. The building prototype has been defined and characterized as reported in Ref. [14]. The energy efficiency measures considered are related to envelope, appliances, and systems. Electricity and gas savings derived from the implementation of the different efficiency measures are calculated by carrying out energy simulations using the BeOpt tool [15] and making a comparison with the baseline building as explained in Ref. [16]. For each measure, data on costs, lifetime, payback, embodied energy, electricity and gas savings are collected or calculated for different energy efficiency measures as reported in the Excel spreadsheet attached to this paper.

## 2 Experimental design, materials and methods

The experimental design of the study was to evaluate how embodied as well as site energy including associated emissions can be minimized in a residential building using a multi-criteria approach. For methods, we had previously developed very detailed information on typical residential housing characteristics and physical parameters with which to base a simulation analysis [1]. This included the physical and performance characteristics of each evaluated option as well as cost data that were assembled. However, a key additional need for the multi-criteria approach was to obtain valid data on the embodied energy. These data were evaluated by thoroughly examining previous research relative to the identified options and then to render them into the proper units. The collected information was then added to the database [[2], [3], [4], [5], [6], [7], [8], [9], [10]]. This allowed each option to be simulated in detail providing both changes in embodied and operational energy use as well as associated emissions. The simulation analysis, while confined to a residential prototype, was rigorous and is covered in detail in the source publication [1]. The final data set used in reproduced here. The interpretation of each column (A-AP) of the spreadsheet is explained below:

• A: Alpha-numeric code of simulated energy efficiency measures;
• B: Energy efficiency measure category (appliance, lighting, equipment, heating, cooling, ventilation, water heating, insulation, ceiling, walls, exterior, windows, air leakage, photovoltaic system);
• C: Description of the technology: details of the equipment, appliances, materials, thicknesses and associated parameters;
• D: Estimated measure initial cost (€);
• E: Incremental cost (€);
• F: Lifetime of the measure (yr);
• G: Number of the measure for a residential building (25 fixtures for lighting);
• H: Area of the measure (m2, where applicable);
• I: Thickness of the measure (cm, where applicable);
• J: Volume of the materials (m3, where applicable);
• K: Material description comprising the measure;
• L: Density of the measure (kg/m^3^, 1 for items);
• M: Weight of the measure (kg), which is the product of the density and volume;
• N: the MJ per kg for the measure or per unit (MJ/kg/Unit), embodied energy [[2], [3], [4], [5], [6], [7], [8], [9], [10], [11], [12], [13], [14], [15], [16], [17], [18]];
• O: Computed total embodied energy (MJ) of the measure; from tables or data for items, computed for area and volume based measures;
• P: Embodied Energy (kWh), the total embodied energy in Column O (MJ) translated into kWh;
• Q: Annual Electricity Save (kWh), the simulated annual electricity savings of the measure;
• R: Annual Gas Save (kWh), the simulated annual natural gas savings of the measure;
• S: Annual CO_2_ Reduction Emissions (kg/yr): annual electricity savings translated into its average annual impact (0.447 kg/kWh for electricity and 0.181/kWh for natural gas) including site to source conversion factors for electricity;
• T: Annual Total Save (€), the annual energy monetary savings given the reductions in Col. Q for electricity and R (gas) times their average cost (AL and AL, respectively);
• U: Lifetime Electric Save (kWh), electricity savings over the life of the involved measure; Column Q * Col. F;
• V: Lifetime Gas Save (kWh), natural gas savings over the life of the involved measure: Column R * Column F;
• W: Lifetime Electricity Save (€), electricity monetary savings over the life of the involved measure;
• X: Lifetime Gas Save (€): natural gas monetary savings over the life of the involved measure;
• Y: Total Lifetime Save (€): total energy monetary savings over the life of the involved measure;
• Z: Simple Payback (yr), total initial cost of the measure (Column E) divided by total annual energy savings (Column T);
• AA: Differential Series Present Worth Factor. This numeric factor translates a stream of future energy savings into their present worth in year zero based on the nominal discount rate (AN), real discount rate (AP), the energy escalation rate (AN), general inflation rate (AP) and number of years which the measure (Column F) will last;
• AB: Net Present Value Energy Save (€), Net present value of energy savings. Column T times Column AA minus Total Cost (Column E);
• AC: Block wall volume (m^3^), volume of block walls: assumes 15.24 cm block times Column H;
• AD: Block (kg/m^3^), density of block wall construction (kg/m^3^); depend on block cores;
• AE: E.E. Block (MJ/kg), embodied energy per kg of block wall;
• AF: E.E Block (kWh), embodied energy of block wall;
• AG: Frame (MJ/m^2^), embodied energy of frame wall depending on construction type and characteristics;
• AH: E.E. Frame (kWh): embodied energy of frame wall in kWh (Col.H * AG/3.6);
• AI: Simulated (kWh/yr): simulated kWh per year for each case (operational energy);
• AI: Simulated (Therms/yr): simulated natural gas consumption for each case in Therms (29.3 kWh/Therm);
• AL: Average electric price (€/kWh): 0.250 €/kWh;
• AL: Average natural gas price (€/kWh): 0.058 €/kWh;
• AN: Nom. Discount Rate: nominal fractional discount rate (includes inflation; 0.05);
• AN: Energy Escalation Rate: nominal fractional energy escalation rate (includes inflation; 0.05);
• AP: Inflation Rate, general inflation rate (fractional, includes inflation; 0.02);
• AP: Real Discount Rate, real discount rate (fractional, not including inflation; 0.03).

In the spreadsheet, the base case building options (reference case described in Ref. [14]) are marked in bold. The column color shading indicates optimization points. In details, orange: embodied energy and CO_2_ emissions (which we aim to minimize); light blue: life time operational energy savings (which we aim at maximize); green: initial cost, simple payback (which we aim at minimize), net present value of savings (which we aimed to maximize). The approach to select the measures to be implemented in the building prototype from the results shown here is based on the multi-criteria optimization methods fully described in the source publication [1]. Uncertainties in associated parameters and assumptions were also outlined in detail in the specific analysis.
